# Team Coaching to Support Nursing Home Implementation of Complex Psychosocial Interventions

**DOI:** 10.1093/geroni/igaf122.314

**Published:** 2025-12-31

**Authors:** A Lynn Snow, Megan McCullough, Michele Koziel, Lindsay Pack, Kelly Presser, Selma Stovrag, Alex Wong, Christine Hartmann

**Affiliations:** The University of Alabama, Tuscaloosa, Alabama, United States; University of Massachusetts Lowell, Lowell, Massachusetts, United States; Geriatrics and Extended Care, VA Central Office, Washington, District of Columbia, United States; Geriatrics and Extended Care, VA Central Office, Washington, District of Columbia, United States; Geriatrics and Extended Care, VA Central Office, Washington, District of Columbia, United States; Geriatrics and Extended Care, VA Central Office, Washington, District of Columbia, United States; Geriatrics and Extended Care, VA Central Office, Washington, District of Columbia, United States; University of Massachusetts Lowell, Lowell, Massachusetts, United States

## Abstract

We describe the use of team coaching to support nursing home implementation across two clinical trials of a front-line centered team huddling intervention, one focused on improving sleep for people with dementia and one focused on improving mobility and reducing falls in nursing home residents. Team coaching is a sub-type of practice facilitation in which the focus is on the entity of the team as well as the individuals within the team, and their relationships, communications, processes, goals, and outcomes with their stakeholders (e.g., employees, residents, families, and corporate) as well as each other. The first trial has completed intervention in 11 nursing homes (of 24 total) and the second trial’s intervention is near completion in 8 homes. Team coaching is provided by a group of coaches with nursing home and team coaching experience (including nurses, social workers, a nursing home administrator and organizational development experts). Team coaching supervision is provided by experienced coaches. Regular qualitative interviews with the teams and coaches were analyzed for relevant themes related to program implementation and the benefits and challenges of team coaching. Preliminary emergent themes from the teams include reflections on the ways in which team coaching has been helpful and emerging challenges. Preliminary emergent themes from the coaches include identification of typical “turning points” that indicate the team has begun to internalize the principles and practices of the intervention, typical “pain points” that challenge the progress of the team, and challenges and opportunities in supporting team development.

